# Longitudinal imaging of individual olfactory sensory neurons *in situ*

**DOI:** 10.3389/fncel.2022.946816

**Published:** 2022-07-22

**Authors:** Joseph D. Zak

**Affiliations:** Department of Biological Sciences, University of Illinois at Chicago, Chicago, IL, United States

**Keywords:** olfactory, olfaction, *in vivo* imaging, olfactory sensory neuron (OSN), protocol

## Abstract

Olfactory sensory neurons are found deep within the nasal cavity at a spatially restricted sheet of sensory epithelium. Due to their location behind the nasal turbinates, accessing these cells for physiological measurements in living animals is challenging, and until recently, not possible. As a further complication, damage to the overlying bone on the dorsal surface of the snout disrupts the negative pressure distribution throughout the nasal cavities, which fundamentally alters how odorants are delivered to the sensory epithelium and the inherent mechanosensory properties of olfactory sensory neurons in live animals. The approach described here circumvents these limitations and allows for optical access to olfactory sensory neurons in mice across time scales ranging from days to months.

## Introduction

**FIGURE 1:**
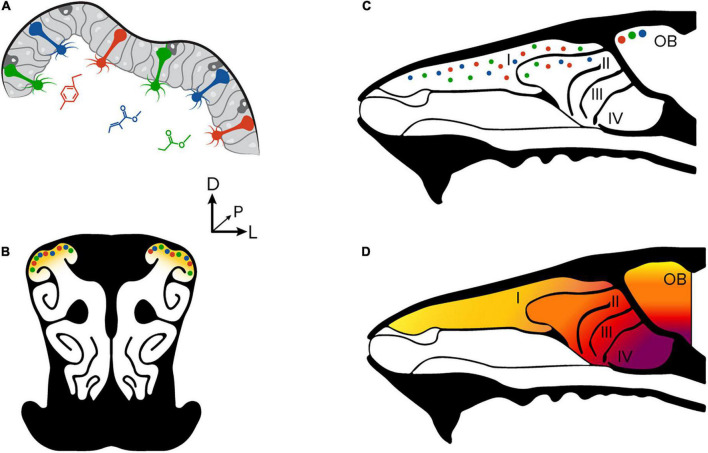
Anatomical organization of the olfactory periphery. **(A)** Olfactory sensory neurons in the olfactory epithelium. Each sensory neuron is sensitive to a unique molecular feature of an odorant molecule. **(B)** A coronal section through the olfactory epithelium and nasal turbinates of a mouse. A large proportion of sensory neurons are found in the dorsal aspect of the turbinates. **(C)** A sagittal section at the midline through the nasal cavity and olfactory bulb. Olfactory sensory neurons found in the dorsal epithelium project to glomeruli on the dorsal surface of the olfactory bulb. Modified from Zak et al. (2020). **(D)** The zonal organization of the olfactory epithelium is consistent with glomerular placement in the olfactory bulb. The OSN subtypes found in the dorsal epithelium project their axons to the dorsal olfactory bulb.

Olfactory transduction begins when volatilized odorant molecules are inhaled through the nasal cavities, where they bind to olfactory receptor proteins that are found on the cilia of olfactory sensory neurons (OSNs). The mouse olfactory epithelium contains ∼10^7^ individual OSNs that can be broadly classified into one of ∼1,100 subtypes based on their receptor protein expression (Zhang and Firestein, 2002; Kawagishi et al., 2014). Each OSN expresses a single receptor protein subtype that is selective to a chemical functional group on an odorant molecule (Figures 1A,B). Odorant molecules each activate a combinatorial pattern of OSNs depending on their ligand sensitivity, which then signals the presence of functional groups in the environment.

All OSN that express the same receptor protein project an axon toward one of two common structures on the surface of the olfactory bulb called glomeruli (Mombaerts, 2006; Francia and Lodovichi, 2021). The distribution of olfactory sensory neuron subtypes in the dorsal aspect of the olfactory epithelium is consistent with the patterning of glomeruli on the dorsal surface of the olfactory bulb (Figures 1C,D; Ressler et al., 1993; Murthy, 2011). Many studies have measured the collective activity of olfactory sensory neurons to OSNs at their axon terminals because the approach provides an excellent signal-to-noise ratio and population readout (Meister and Bonhoeffer, 2001; Wachowiak and Cohen, 2001; Fletcher et al., 2009; Soucy et al., 2009; Albeanu et al., 2018; Zak et al., 2018); however, the ability to measure stimulus encoding properties at the single-cell level is not possible due to the tight packing of sensory neuron axons within the glomerular neuropil. Other studies of OSN function and physiology have avoided these limitations by excising tissue for *ex vivo* preparations (Ma et al., 1999; Ma and Shepherd, 2000; Xu et al., 2020), or by recording from a limited number of cells by puncturing the overlying bone (Duchamp-Viret et al., 1999). Both approaches are incompatible with measuring neural responses in the context of natural respiration or at the single-cell level.

This protocol describes an adapted experimental preparation that allows for repeated *in vivo* measurements of sensory neuron activity in freely breathing animals. The approach combines a thinned-skull technique (Yang et al., 2010) with chronic coverslip implantation that allows for clear visualization of neuronal responses on a scale of weeks to months. The preparation is best suited for multiphoton imaging due to the deep embedding of sensory neurons in the olfactory epithelium.

## Methods

### Experimental model and subject details

Adult (>8 weeks) OMP-GCaMP3 mice of both sexes were used in this study. Three separate animals were used for epithelial windows and the collection of example data. Mice were acquired from in-house breeding stocks and maintained within Harvard University’s Biological Research Infrastructure for the duration of the study. All animals were between 20 and 30 g before surgery and singly housed following any surgical procedure. All mice used in this study were housed in an inverted 12-h light cycle at 22 ± 1°C at 30–70% humidity and fed *ad libitum*. All the experiments were performed under the guidelines set by the National Institutes of Health and approved by the Institutional Animal Care and Use Committee at Harvard University. Thinned-bone cranial windows were prepared as described below and animals were allowed to recover for at least 3 days before the initiation of imaging experiments.

### Multiphoton imaging

A custom-built two-photon microscope was used for *in vivo* imaging. Fluorophores were excited and imaged with a water immersion objective (20X, 0.95 NA, Olympus) at 920 nm using a Ti:Sapphire laser (Mai Tai HP, Spectra-Physics). Images were acquired at 16-bit resolution and 4–8 frames/s. Fields of view ranged from 180 × 180 μm to 720 × 720 μm. The point-spread function of the microscope was measured to be 0.51 × 0.48 × 2.12 μm. Image acquisition and scanning were controlled by custom-written software in LabVIEW (National Instruments). Emitted light was routed through two dichroic mirrors (680dcxr, Chroma, and FF555-Di02, Semrock) and collected by a photomultiplier tube (R3896, Hamamatsu).

### Odor stimulation

The monomolecular odorant Methyl tiglate (Penta Manufacturing) was used as a stimulus and delivered by a custom-built 16-channel olfactometer that was controlled by custom-written software in LabVIEW. The initial concentration of each odor was 16% (v/v) in mineral oil then further diluted 16 times with air. For all experiments, the airflow to the animal was held constant at 100 mL/min and odors were injected into a carrier stream. Respiration signals were collected using an airflow sensor (Honeywell AWM3300V; Bolding and Franks, 2017; Grimaud and Murthy, 2018).

### Data analysis

Images were processed using both custom and available MATLAB (MathWorks) scripts. Motion artifact compensation and denoising were done using NoRMCorre (Pnevmatikakis and Giovannucci, 2017). The CaIMaN CNMF pipeline (Pnevmatikakis et al., 2016) was used to select and demix ROIs. ROIs were further filtered by size and shape to remove merged cells. dF/F values were calculated using the mean of a baseline period of at least 20 frames. To identify OSNs that were significantly odorant-modulated, three points centered on the peak dF/F signal after odorant delivery were averaged. OSNs were classified as significantly odorant-modulated if their peak averaged response exceeded 2.5 standard deviations of the baseline noise.

### Surgical procedures

Cranial windows are prepared over a 2-day period. The first day consists of collecting necessary equipment and preparing the animal for surgery. The main surgery can typically be completed in 60–90 min the following day.

### Day prior to surgery

1. Make a silicone whip by dipping an old drill bit in silicone sealant and withdrawing to leave a taper, as described by Shih et al. (2012).
2. Make coverslips. Using a diamond-tipped scribe, score a large coverslip in a checkerboard yielding rectangles 3 mm × 2 mm. From the checkerboard, break off individual sections to be used. Store in ethanol overnight. The remaining coverslip sections can be saved for future use.
3. Give the experimental animal a single dose of carprofen (5 mg/kg) subcutaneously to help prevent inflammation during surgery and recovery.

### Day of surgery

**FIGURE 2:**
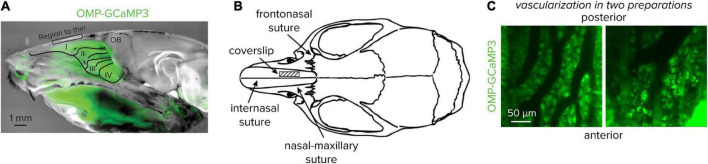
Location of the thinned bone and coverslip placement. **(A)** A cross-section at the midline of an *OMP-GCaMP3* mouse. Olfactory marker protein expression can be observed directly under the area to thin. **(B)** Location of the coverslip placement. The thinned bone and coverslip should be placed directly anterior to the frontonasal suture, between the internasal suture and nasal-maxillary suture. **(C)** Two-photon images of the dense vascularization of the olfactory epithelium and tight packing of sensory neurons from two separate preparations.

1. One hour prior to surgery, give the animal a second dose of carprofen, (5 mg/kg) subcutaneously.
2. Collect consumables (Table 1) and sterilize instruments (Table 2) where possible using a steam autoclave or hot bead sterilizer.
3. Cut small corners of gelfoam from a larger sheet and place them in 1 mL of artificial cerebrospinal fluid (aCSF).
4. Sterilize the surgical area and stereotax with ethanol and cover the area with a drape.
5. Anesthetize the animal using a mixture of ketamine and xylazine (100 and 5 mg/kg, respectively). Before proceeding, verify anesthesia depth by toe pinch.
6. Place petroleum jelly on the eyes to prevent them from drying out.
7. Remove the hair from the crown of the head to the tip of the snout using electric clippers. Use an ethanol pad to clear any remaining hair or debris.
8. Place the animal in the stereotaxic device and ensure that the head is held firmly in place by ear bars by gently pressing on the snout.
9. Use the scalpel blade to make an incision at the midline from the crown of the head to the nares. Do not cut the tissue surrounding the nares.
10. Remove the flaps of skin on either side of the midline cut from the crown of the head toward the ocular sinus, then to a position just posterior of the nares. Take special care not to damage arteries that surround the eyes when removing this skin. If bleeding occurs, use small pieces of gelfoam soaked in aCSF to induce clotting and cellulose spears to absorb any remaining fluids.
11. Use a cotton-tipped applicator to remove any fascia or connective tissue covering the skull and snout of the animal. A scalpel blade may be used on older animals where more tissue is present.
12. Place a small drop of aCSF on the bone covering the snout in the area to be thinned (see Figures 2A,B). The thinned area should be directly anterior to the frontonasal suture, between the internasal suture and nasal-maxillary suture where the bone plate begins to widen. Allow the aCSF to soak in and soften the bone for 1–2 min.
13. Under the stereoscope, with a fresh drill bit, begin to thin the bone over the olfactory epithelium by lightly pressing the bit to the skull and moving in slow circular motions.
14. Periodically, use cellulose spears and more aCSF to clean the thinned area.
15. Check the thinning progress by placing a small amount of aCSF on the thinned area. If the bone remains opaque when moistened, continue with the drill bit. If the bone is transparent, place a fresh blade on the scalpel.
16. In sweeping motions from posterior to anterior, use the scalpel blade to gently shave the remaining bone.
17. Continue shaving the bone until it becomes soft and flexible like cellophane. Often, when the bone has reached the desired degree of thinness, the bone will begin to form a rounded upward projecting bubble from pressure within the nasal cavity. Special care needs to be taken not to puncture the thinned bone. Punctured bone will ruin the preparation by affecting airflow through the nasal cavity and allowing the glue to be absorbed below the bone in subsequent steps.
18. Clean the thinned portion of the skull with aCSF and remove any debris.
19. Create a slurry by mixing a small amount of tin oxide and aCSF on the thinned section of bone.
20. Use the silicone whip drill bit to polish the thinned portion of the skull with the tin oxide slurry, again taking care not to damage the thinned section. The polishing technique smooths the bone surface and provides a tight junction for the coverslip (step 22), while also reducing the bone opacity.
21. Again, clean the skull and remove any debris. Allow the skull to dry completely before moving to the next step.
22. Place a drop of cyanoacrylate on the thinned portion of the skull and place one of the coverslips on the drop of glue. The cyanoacrylate should spread evenly under the coverslip and reach the edges. Allow the cyanoacrylate to dry completely.
23. Use cyanoacrylate to affix the head bar to the crown of the skull and allow it to dry completely.
24. Mix dental cement according to instructions. Use a plastic pick to spread the dental cement to cover the headbar and the remaining portions of the exposed skull. If a water immersion objective will be used in imaging experiments, use the cement to build a well around the edges of the coverslip, taking care not to cover the nares or the snout of the animal.
25. Once the animal has returned to a sternal and upright position, administer analgesics. Allow the animal to recover for at least 24 h.

**TABLE 1:** Consumable products.

Item	Vendor	Catalog number
Silicone sealant	VWR	470021-514
Petroleum jelly	VWR	76337-102
Scalpel blade	VWR	21909-610
Cellulose spears	Henry Schein	1355297
PBS (aCSF)	VWR	45000-446
Cyanoacrylate glue	VWR	19807-542
Cotton-tipped applicator	VWR	89176-684
Plastic pick dental cement	VWR	470020-502
Tin oxide powder	Sigma-Aldrich	549657
Gel foam	Henry Schein	9083300
Dental cement	Parkell	S380
Coverslips (1.5)	VWR	16004-302
Ketamine	Patterson Veterinary	07-803-6637
Xylazine	Patterson Veterinary	07-808-1939
Carprofen	Patterson Veterinary	07-844-7425
Buprenorphine SR-Lab	ZooPharm	–

**TABLE 2:** Equipment.

Item	Vendor	Catalog number
Hair clippers	Stoelting	51465
Scalpel holder	Stoelting	52171
Fine scissors	Stoelting	52130-50
Micro drill	World Precision Instruments	503598
Drill bit × 2	Stoelting	514551
Diamond scribe	VWR	52865-005
Stereotax	Stoelting	51625
Stereoscope	AmScope	SM-4B
Light source	AmScope	LED-14M

While this preparation can be used for acute experiments immediately following window implantation, for best results, animals should recover for 1–3 days. During the surgical procedures, fluids can be ingested into the nasal cavities which can damage OSNs or occlude the ability to observe their functional responses. Allowing fluid to disperse and inflammation to subside improves calcium signal acquisition.

## Results

Following a recovery period, both structural and functional fluorescence signals can be collected from the olfactory epithelium using 2-photon microscopy. The olfactory epithelium is highly vascularized, which will help in approximating the appropriate imaging depth, as well as, repeatedly finding the same imaging location across experimental days (Figure 2C). Importantly, care needs to be taken to distinguish between the OSN dendritic knob, found at the surface of the epithelium and the soma found deeper within the tissue. The transduction current in OSNs is partially carried by calcium and it is not known if the calcium dynamics at the dendritic knob are representative of spike-mediated calcium signals that propagate from the axon near the soma. In the dorsal epithelium, the orientation of OSNs is inverted, therefore the dendritic knobs will be found at a deeper imaging plane than the somata. Individual neurons can be visualized at rest; however, OSNs have variable spontaneous activity under baseline conditions, thereby limiting resting fluorescence signals from functional indicators if the spontaneous activity is low (Reisert, 2010; Connelly et al., 2013). Brief odor pulses can be delivered to drive OSN activity and aid in optimizing the imaging plane.

Once an optimal plane has been identified, the functional responses of dozens to hundreds of OSNs can be simultaneously measured. The density of neural activity in the olfactory epithelium varies as a function of odorant identity (Zak et al., 2020). If odorants that generate dense responses are used, care should be taken to account for background signals that may contaminate responses at individual cells. For all example experiments, the odorant Methyl tiglate was selected due to the high density of neural responses that it generates and for consistency across experiments.

### Responses from individual OSNs in the olfactory epithelium

**FIGURE 3:**
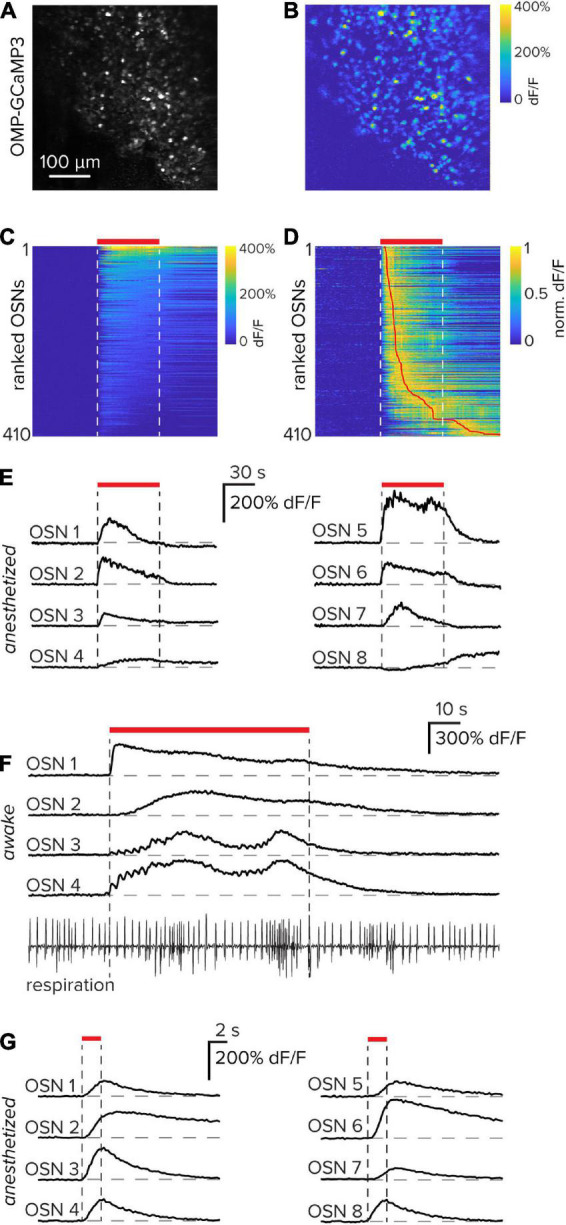
Functional responses of olfactory sensory neurons through an implanted window. **(A)** Resting fluorescence image of olfactory sensory neurons expressing GCaMP3 in the olfactory epithelium. **(B)** Functional response of the entire imaging field to the odorant Methyl tiglate in an anesthetized mouse. **(C)** Calcium responses of 410 individual sensory neurons from the images in parts **(A,B)** that were significantly stimulus modulated. Vertical white dashed lines and horizontal red bar indicate the odorant delivery period. Data are sorted by their mean response in the odorant period. **(D)** Data in part **(C)** normalized to their peak and sorted by the latency to reach their peak. The vertical red line indicates the peak location. **(E)** Eight example sensory neuron responses collected from parts **(A–D)**. **(F)** Example functional responses collected through a thinned bone window in an awake mouse. Responses to individual respiration events, as well as respiration-frequency modulation, can be observed in some OSNs. In the respiration trace at the bottom, upward is inhalation. **(G)** Eight example OSN functional responses from the same imaging field using brief 2 s odorant pulses in an anesthetized mouse.

To capture functional responses from OSNs within the olfactory epithelium, 60-s pulses of the odorant Methyl tiglate were delivered to an anesthetized, but freely breathing mouse. The imaging session took place 3 days after the bone-thinning procedure to allow for surgically induced inflammation to subside. From a single imaging field, 410 OSNs (Figure 3A) that were significantly odorant-modulated were identified (Figures 3B–D). OSNs were classified as significantly odorant-modulated if their peak response exceeded 2.5 standard deviations of the baseline noise. Although each of the OSNs had a clear stimulus-response, the waveform and response latency varied from cell to cell suggesting that multiple OSN subtypes are present in the imaging field (Figure 3E). With a diverse panel of odorants, populations of cells that express the same odorant receptor proteins could in principle be identified by their odorant tuning.

Many of these experiments were performed in anesthetized animals to exclude confounds associated with variable sniffing rates during a stimulus sampling period; however, it is also possible to perform similar experiments in awake animals. In these instances, functional OSN responses are locked to individual respiration cycles. Furthermore, when animals initiate sniffing bouts as indicated by a transient increase in respiration frequency, OSN responses are often directly modulated (Figure 3F).

Odorant stimulations of 60 s give rise to the possibility of adaptation both at the level of receptor-ligand dynamics and intracellular calcium handling. To limit these potential effects, odorants were also delivered at a more physiologically relevant time scale of 2 s. Even at these brief stimulus durations, robust and repeatable OSN functional responses were observed (Figure 3G). Together, these example data demonstrate that functional responses of OSNs can be reliably obtained using a thinned-bone procedure in combination with chronic window implantation.

### Functional responses and window viability persists for weeks

**FIGURE 4:**
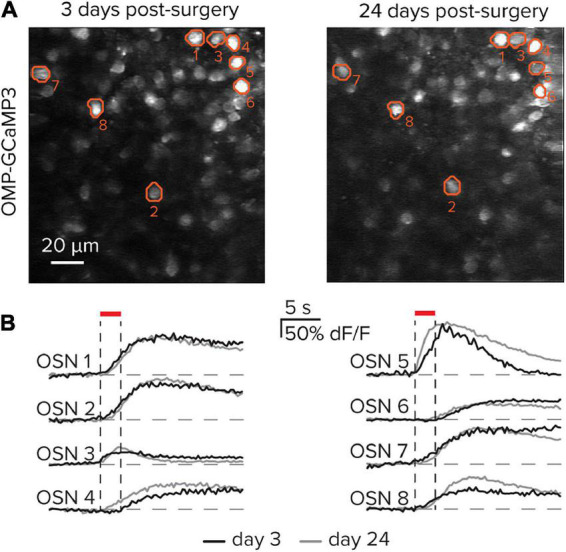
Longitudinal imaging of olfactory sensory neurons. **(A)** The same imaging field in the olfactory epithelium 21 days apart. A subset of eight cells is identified in each image. **(B)** Functional responses from the eight identified cells in part **(A)** on post-surgical days 3 and 24.

To date, several studies have used a thinned-boned procedure to capture functional responses in OSNs (Iwata et al., 2017; Inagaki et al., 2020; Zak et al., 2020). A key advance here includes surgical implantation of a coverslip above the thinned bone, which allows the same imaging field to be longitudinally followed across experimental days. To demonstrate the feasibility of repeatedly imaging the same subregion of the olfactory epithelium, the protocol above was used to create a stable chronic window. After allowing the window to stabilize for 3 days following implantation, the same subset of OSNs was imaged and their functional responses to the odorant Methyl tiglate were evaluated at time points spread across 3 weeks (Figure 4A). The same populations of OSNs can be observed in the resting fluorescence images, while their functional responses are stable between imaging sessions (Figure 4B). While the examples here demonstrate the ability to capture matched OSN responses across 21 days, the stability and clarity of the window can remain viable for several months, thereby allowing for extended imaging protocols.

## Discussion

The approach described here has several advantages over acute preparations that allow for optical access to the olfactory epithelium (Iwata et al., 2017; Inagaki et al., 2020, 2021). First, the activity of OSNs can be measured longitudinally, allowing for matched comparisons across experimental conditions. Importantly, the application of cyanoacrylate under the glass coverslip prevents bone regrowth and thickening thereby allowing preparations to remain viable for experiments that can range from weeks to months. Furthermore, animals need not be anesthetized during imaging following a recovery period. This allows OSN activity to be measured within the context of natural respiration and sniffing dynamics in behaving animals. Combining behavioral readouts with measurements of the functional properties of individual neurons at the sensory periphery holds promise to provide new insight into sensory coding and computation at the earliest stages of the olfactory system.

There are, however, a few limitations that should be noted. Due to the conserved zonal organization of the olfactory epithelium and the olfactory bulb (Ressler et al., 1993), there does not appear to be a substantial increase in the number of OSN subtypes available (Zak et al., 2020). Because the olfactory epithelium is organized on a dorsal-ventral axis with respect to OSN subtypes, many remain inaccessible (Figure 1B). From a technical perspective, the increased number of steps and the need to polish the thinned section of bone to maintain clarity creates more opportunities to damage the experimental preparation thereby potentially slowing workflows.

By implementing this approach, several challenging questions in sensory neurobiology can now be addressed. First transient disruptions of OSN activity profiles, like those imparted by the SARS-CoV-2 virus, can now be observed over the course of treatments or other manipulations to the supporting sustentacular cells which are affected by the virus. Furthermore, recent studies provide evidence of efferent modulation of the olfactory epithelium (Zhou et al., 2022), the experimental preparation described here may prove key to understanding neuromodulatory effects on peripheral sensory coding. Finally, the highly regenerative processes of the olfactory epithelium can be measured both in terms of sensory neuron turnover, but also changes in sensory coding properties with respect to the stimulus environment.

## Data availability statement

The raw data supporting the conclusions of this article will be made available by the authors, without undue reservation.

## Ethics statement

The animal study was reviewed and approved by the Institutional Animal Care and Use Committee at Harvard University.

## Author contributions

JZ designed the protocol, acquired and analyzed the data, and wrote the manuscript.

## Conflict of interest

The author declares that the research was conducted in the absence of any commercial or financial relationships that could be construed as a potential conflict of interest.

## Publisher’s note

All claims expressed in this article are solely those of the authors and do not necessarily represent those of their affiliated organizations, or those of the publisher, the editors and the reviewers. Any product that may be evaluated in this article, or claim that may be made by its manufacturer, is not guaranteed or endorsed by the publisher.

## References

[B1] Albeanu D. F. Provost A. C. Agarwal P. Soucy E. R. Zak J. D. Murthy V. N. (2018). Olfactory marker protein (OMP) regulates formation and refinement of the olfactory glomerular map. *Nat. Commun.* 9:5073. 10.1038/s41467-018-07544-9 30498219 PMC6265328

[B2] Bolding K. A. Franks K. M. (2017). Complementary codes for odor identity and intensity in olfactory cortex. *Elife* 6:e22630. 10.7554/eLife.22630.023 28379135 PMC5438247

[B3] Connelly T. Savigner A. Ma M. (2013). Spontaneous and sensory-evoked activity in mouse olfactory sensory neurons with defined odorant receptors. *J. Neurophysiol.* 110 55–62. 10.1152/jn.00910.2012 23596334 PMC3727041

[B4] Duchamp-Viret P. Chaput M. A. Duchamp A. (1999). Odor response properties of rat olfactory receptor neurons. *Science* 284 2171–2179. 10.1126/science.284.5423.2171 10381881

[B5] Fletcher M. L. Masurkar A. V. Xing J. Imamura F. Xiong W. Nagayama S. (2009). Optical imaging of postsynaptic odor representation in the glomerular layer of the mouse olfactory bulb. *J. Neurophysiol.* 102 817–830. 10.1152/jn.00020.2009 19474178 PMC2724327

[B6] Francia S. Lodovichi C. (2021). The role of the odorant receptors in the formation of the sensory map. *BMC Biol.* 19 1–18. 10.1186/s12915-021-01116-y 34452614 PMC8394594

[B7] Grimaud J. Murthy V. N. (2018). How to monitor breathing in laboratory rodents: a review of the current methods. *J. Neurophysiol.* 120 624–632. 10.1152/jn.00708.2017 29790839 PMC6139454

[B8] Inagaki S. Iwata R. Imai T. (2021). *In vivo* Optical Access to Olfactory Sensory Neurons in the Mouse Olfactory Epithelium. *Bio-Protocol.* 11:e4055. 10.21769/BioProtoc.4055 34262998 PMC8260259

[B9] Inagaki S. Iwata R. Iwamoto M. Imai T. (2020). Widespread inhibition, antagonism, and synergy in mouse olfactory sensory neurons *in vivo*. *Cell Rep.* 31:107814. 10.1016/j.celrep.2020.107814 32610120

[B10] Iwata R. Kiyonari H. Imai T. (2017). Mechanosensory-based phase coding of odor identity in the olfactory bulb. *Neuron* 96 1139–1152e1137. 10.1016/j.neuron.2017.11.008 29216451

[B11] Kawagishi K. Ando M. Yokouchi K. Sumitomo N. Karasawa M. Fukushima N. (2014). Stereological quantification of olfactory receptor neurons in mice. *Neuroscience* 272 29–33. 10.1016/j.neuroscience.2014.04.050 24797329

[B12] Ma M. Chen W. R. Shepherd G. M. (1999). Electrophysiological characterization of rat and mouse olfactory receptor neurons from an intact epithelial preparation. *J. Neurosci. Methods* 92 31–40. 10.1016/S0165-0270(99)00089-8 10595701

[B13] Ma M. Shepherd G. M. (2000). Functional mosaic organization of mouse olfactory receptor neurons. *Proc. Natl. Acad. Sci. U S A* 97 12869–12874. 10.1073/pnas.220301797 11050155 PMC18856

[B14] Meister M. Bonhoeffer T. (2001). Tuning and topography in an odor map on the rat olfactory bulb. *J. Neurosci.* 21 1351–1360. 10.1523/JNEUROSCI.21-04-01351.2001 11160406 PMC6762249

[B15] Mombaerts P. (2006). Axonal Wiring in the Mouse Olfactory System. *Annu. Rev. Cell Dev. Biol.* 22 713–737. 10.1146/annurev.cellbio.21.012804.093915 17029582

[B16] Murthy V. N. (2011). Olfactory Maps in the Brain. *Annu. Rev. Neurosci.* 34 233–258. 10.1146/annurev-neuro-061010-113738 21692659

[B17] Pnevmatikakis E. A. Giovannucci A. (2017). NoRMCorre: an online algorithm for piecewise rigid motion correction of calcium imaging data. *J. Neurosci. Methods* 291 83–94. 10.1016/j.jneumeth.2017.07.031 28782629

[B18] Pnevmatikakis E. A. Soudry D. Gao Y. Machado T. A. Merel J. Pfau D. (2016). Simultaneous denoising, deconvolution, and demixing of calcium imaging data. *Neuron* 89 285–299. 10.1016/j.neuron.2015.11.037 26774160 PMC4881387

[B19] Reisert J. (2010). Origin of basal activity in mammalian olfactory receptor neurons. *J. Gen. Physiol.* 136:540. 10.1085/jgp.201010528 PMC2964517 20974772

[B20] Ressler K. J. Sullivan S. L. Buck L. B. (1993). A zonal organization of odorant receptor gene expression in the olfactory epithelium. *Cell* 73 597–609. 10.1016/0092-8674(93)90145-G 7683976

[B21] Shih A. Y. Mateo C. Drew P. J. Tsai P. S. Kleinfeld D. (2012). A polished and reinforced thinned-skull window for long-term imaging of the mouse brain. *J. Vis. Exp*. 2012:3742. 10.3791/3742 22433225 PMC3460568

[B22] Soucy E. R. Albeanu D. F. Fantana A. L. Murthy V. N. Meister M. (2009). Precision and diversity in an odor map on the olfactory bulb. *Nat. Neurosci.* 12 210–220. 19151709 10.1038/nn.2262

[B23] Wachowiak M. Cohen L. B. (2001). Representation of odorants by receptor neuron input to the mouse olfactory bulb. *Neuron* 32 723–735. 10.1016/s0896-6273(01)00506-2 11719211

[B24] Xu L. Li W. Voleti V. Zou D. J. Hillman E. M. C. Firestein S. (2020). Widespread receptor-driven modulation in peripheral olfactory coding. *Science* 368:eaaz5390. 10.1126/science.aaz5390 32273438 PMC7443284

[B25] Yang G. Pan F. Parkhurst C. N. Grutzendler J. Gan W. B. (2010). Thinned-skull cranial window technique for long-term imaging of the cortex in live mice. *Nat. Protoc.* 5 201–208. 10.1038/nprot.2009.222 20134419 PMC4690457

[B26] Zak J. D. Grimaud J. Li R. C. Lin C. C. Murthy V. N. (2018). Calcium-activated chloride channels clamp odor-evoked spike activity in olfactory receptor neurons. *Sci. Rep.* 8 1–13. 10.1038/s41598-018-28855-3 30006552 PMC6045664

[B27] Zak J. D. Reddy G. Vergassola M. Murthy V. N. (2020). Antagonistic odor interactions in olfactory sensory neurons are widespread in freely breathing mice. *Nat. Commun.* 11:3350. 10.1038/s41467-020-17124-5 32620767 PMC7335155

[B28] Zhang X. Firestein S. (2002). The olfactory receptor gene superfamily of the mouse. *Nat. Neurosci.* 5 124–133. 10.1038/nn800 11802173

[B29] Zhou H. Q. Zhuang L. J. Bao H. Q. Li S. J. Dai F. Y. Wang P. (2022). Olfactory regulation by dopamine and DRD2 receptor in the nose. *Proc. Natl. Acad. Sci. U S A* 119 1–10. 10.1073/pnas.2118570119 35263227 PMC8931335

